# Retraction: Long noncoding RNA-MEG3 contributes to myocardial ischemia–reperfusion injury through suppression of miR-7-5p expression

**DOI:** 10.1042/BSR20190210_RET

**Published:** 2026-08-25

**Authors:** Liyuan Zou, Xiaokun Ma, Shuo Lin, Bingyuan Wu, Yang Chen, Chaoquan Peng

**Affiliations:** 1 Department of Prevention and Health Care, The Third Affiliated Hospital of Sun Yat-sen University, Guangzhou 510630, China; 2 Department of Medical Oncology, The Third Affiliated Hospital of Sun Yat-sen University, Guangzhou 510630, China; 3 Department of Endocrinology and Metabolism, The Third Affiliated Hospital of Sun Yat-sen University, Guangzhou 510630, China; 4 Department of Cardiology, The Third Affiliated Hospital of Sun Yat-sen University, Guangzhou 510630, China

**Keywords:** cardiomyocyte, ischaemia-reperfusion injury, lncRNA MEG3, miR-7-5p

This article is being retracted from *Bioscience Reports* at the request of the Editor-in-Chief and the Editorial Board. This follows an internal investigation that identified high levels of similarity between the Figure 2D model and model + NC panels with the Figure 4F model + shMEG3+inhibitor and model panels respectively. The authors were contacted regarding the concerns but were not able to provide raw data for Figures 2 and 4 as requested. They provided original scans of the western blots, as requested, however not all the blots correlated with the Figures. Replacements were provided for Figures 2 and 4 based off repeat experiments conducted in 2021; however, these were not considered suitable replacements for original data. Given the lack of raw data and inconsistencies between the western blot data provided, the Editorial Board stands by the decision to retract. The authors agree to the retraction.

